# Coexistent faecal incontinence and constipation: A cross-sectional study of 4027 adults undergoing specialist assessment

**DOI:** 10.1016/j.eclinm.2020.100572

**Published:** 2020-10-13

**Authors:** Paul F. Vollebregt, Lukasz Wiklendt, Phil G Dinning, Charles H. Knowles, S.Mark Scott

**Affiliations:** a National Bowel Research Centre and GI Physiology Unit, Blizard Institute, Centre for Neuroscience, Surgery & Trauma, Barts and the London School of Medicine & Dentistry, Queen Mary University of London, London, United Kingdom; b College of Medicine and Public Health, Flinders University, Australia; c Department of Gastroenterology, Flinders Medical Centre, Australia

**Keywords:** Anorectal physiology, Constipation, Faecal incontinence, Pelvic floor dysfunction

## Abstract

**Background:**

In contrast to paediatric and geriatric populations, faecal incontinence and constipation in adults are generally considered separate entities. This may be incorrect.

**Methods:**

Cross-sectional study of consecutive patients (18–80 years) referred to a tertiary unit (2004–2016) for investigation of refractory faecal incontinence and/or constipation and meeting Rome IV core criteria (applied post-hoc) for self-reported symptoms. We sought to determine how frequently both diagnoses coexisted, how frequently coexistent diagnoses were recognised by the referring clinician and to evaluate differences in clinical characteristics between patients with single or both diagnoses.

**Findings:**

Study sample consisted of 4,027 patients (3,370 females [83·7%]). According to Rome IV criteria, 807 (20·0%) patients self-reported faecal incontinence in isolation, 1,569 (39·0%) patients had functional constipation in isolation, and 1,651 (41·0%) met criteria for both diagnoses (coexistent symptoms). In contrast, only 331 (8·2%) patients were referred for coexistent symptoms. Of the 1,651 patients with self-reported coexistent symptoms, only 225 (13·6%) were recognised by the referrer i.e. 86·4% were missed. Coexistent symptoms were most often missed in patients referred for faecal incontinence in isolation. In this group of 1,640 patients, 765 (46·7%) had concomitant symptoms of functional constipation. Opioid usage, comorbidities, childhood bowel problems, mixed incontinence symptoms, prolapse symptoms and structural abnormalities on defaecography were associated with reclassification.

**Interpretation:**

Over 40% of adults referred for anorectal physiological investigation had coexistent diagnoses of faecal incontinence and functional constipation, based on validated criteria. This overlap is overlooked by referrers, poorly documented in current literature, and may impact management.

**Research in context**

**Evidence before this study**

Coexistence of faecal incontinence and constipation is well recognised in both paediatric and geriatric populations. In community-dwelling adults however, these symptoms are generally considered separate entities.

**Added value of this study**

In this study of 4027 consecutive patients referred to a tertiary centre for investigation of refractory faecal incontinence and/or constipation, we found that coexistent symptoms of faecal incontinence and constipation were self-reported in >40% of patients and consistently reported in all age groups and in both sexes, based on internationally-accepted (Rome IV) diagnostic criteria. The overlap in symptoms was replicated using other validated symptom scoring instruments. Symptom coexistence was *not* acknowledged by the referring clinician in 86·4% of patients, suggesting that most clinicians focussed on either constipation or incontinence, rather than both. This is the first study to demonstrate the lack of awareness of coexistent symptoms amongst referring clinicians, which was unchanged during the 12-year study period. Coexistent symptoms were most often missed in patients referred for faecal incontinence. In this group of 1640 patients, 765 (46·7%) also met symptom criteria for a diagnosis of functional constipation. Several demographic factors, symptoms and results of diagnostic investigations were significantly associated with reclassification.

**Implications of all the available evidence**

As in paediatric and geriatric clinical practice, recognition of coexisting symptoms of faecal incontinence and constipation in adult patients can impact specialist management. Therapies such as biofeedback and certain surgical procedures could be better directed to constipation, where faecal incontinence represents a secondary phenomenon.

Alt-text: Unlabelled box

## 1 Introduction

Faecal incontinence and constipation are common symptoms with significant health and societal impacts at all ages [1–5]. Coexistence of these symptoms is well recognised in paediatric and geriatric populations [6]. In paediatric practice, faecal retention associated with stool-withholding behaviour causing ‘overflow’ is regarded as the main cause of faecal incontinence [7]. The prevalence of underlying constipation in children with faecal incontinence attending primary care clinics is reported to be very high (up to 95%) [8]. Conversely, tertiary care studies evaluating treatment of functional constipation have also shown a high proportion of children with concomitant faecal incontinence at baseline, and a subsequent decrease in incontinence episodes at follow-up [9]. Accordingly, faecal incontinence is considered a Rome IV diagnostic criteria for childhood functional constipation [10].

In elderly, faecal impaction is also regarded as a leading cause of faecal incontinence [11]. Reduced mobility, cognitive impairment and side effects of drugs are factors increasing risk of faecal impaction. When complete rectal emptying is achieved in these patients, frequency of faecal incontinence decreases [12].

In contrast to this acknowledged coexistence of faecal incontinence and constipation at both ends of the age spectrum, in adults, these symptoms are frequently considered separate entities. Full journal supplements have been devoted to either faecal incontinence or constipation in isolation, with scant or no reference to the other symptom [13,14]. Likewise, two recent population-based surveys on the prevalence of faecal incontinence and functional constipation were published independently [3,5], and constipation (except hard/lumpy stool) was not evaluated as a risk factor for faecal incontinence [5]. Unlike the Rome IV criteria for functional constipation in children, faecal incontinence is not part of the criteria in adults [1]. This noted, the Rome IV panel in adults have been careful to describe functional bowel disorders as a spectrum of gastrointestinal disorders characterised by predominant symptoms [1]. This acknowledges that there can be considerable overlap between thus-defined syndromes e.g. IBS and functional constipation. Pertinent to this point is the overlap of other symptoms such as diarrhoea which is known as a major risk factor for incontinence [15], [16], [17].

Our own clinical experience in adults indicates that coexistence of faecal incontinence and constipation is frequently overlooked by referrers and may have implications to treatment. On this basis, we sampled a consecutive large series of adults referred for specialist investigation of refractory faecal incontinence and/or constipation who met Rome criteria for faecal incontinence +/- functional constipation [1,4]. Our aims were to:

1. assess how frequently patients met criteria for one or both Rome diagnoses;
2. determine how frequently coexistent diagnoses were recognised by the referring clinician and alternative classification scoring systems;
3. evaluate differences in clinical characteristics between patients with faecal incontinence in isolation, functional constipation in isolation and those with coexistent diagnoses (with a focus on missed functional constipation in patients referred for faecal incontinence).

In subgroup analyses we acknowledge the importance of loose stools as a possible contributor to faecal incontinence.

## 2 Methods

### 2.1 Study sample

Consecutive community-dwelling adults (18–80 years) referred to the Royal London Hospital Gastrointestinal Physiology Unit (period: January 2004-March 2016) were considered for inclusion. Prior to anorectal physiological testing, all patients completed a comprehensive bowel questionnaire (see Supplementary Document) which incorporated internationally-validated symptom classification systems (Rome criteria for faecal incontinence [4] and functional constipation [1], Cleveland Clinic constipation score [18], St Marks incontinence score [19]), other (bowel-related) questionnaires, and a structured evaluation of medical conditions, current medication use and prior surgical/obstetric events.

Subsequently, patients underwent anorectal physiological testing (anorectal manometry, endoanal ultrasonography and rectal sensory testing). The majority of patients also underwent defaecography. Whole-gut transit studies (radio-opaque marker technique) were performed in patients with a history of infrequent defaecation. All tests are described in the Supplementary Document.

### 2.2 Inclusion and exclusion criteria

The study sample was restricted to patients with a minimum dataset of (i) primary reason for referral documented in referral letter; (ii) complete self-reported questionnaires; (iii) anorectal physiology performed. Further patients were excluded if the primary reason for referral was not faecal incontinence or constipation (e.g. isolated prolapse symptoms, anorectal pain). Thereafter, patients had to fulfil Rome IV core criteria for either faecal incontinence (solid/liquid; episodes >monthly) or functional constipation (≥two core symptoms, see Supplementary Document). For consistency, although some patients completed earlier questionnaire iterations (Rome II/III), Rome IV core criteria were applied *post hoc* to all patients irrespective of Rome era.

### 2.3 Self-reported symptoms, diagnosis by referring clinician and alternative questionnaire-based scoring systems

Self-reported symptoms reflected patient responses to (clinical) Rome questionnaires. The primary reason for referral stated in the clinicians’ referral letter was derived by hard copy examination of every clinic letter and categorised as being: (1) faecal incontinence, (2) constipation, or (3) coexistent faecal incontinence and constipation (both being mentioned). Cleveland Clinic constipation (range 0–30) [18] and St Marks incontinence scores (range 0–24) [19] were derived from the bowel questionnaire and widely accepted (gastroenterological and surgical literature) cut-offs applied to make a diagnosis: Cleveland Clinic constipation score (cut off: ≥9); St Marks incontinence score (cut off: ≥6).

### 2.4 Clinical characteristics

Putative risk factors [1,2,4,5] were derived from the structured history and questionnaires and compared between patients with self-reported faecal incontinence in isolation, functional constipation in isolation and coexistent symptoms (Rome IV defined). Further detailed symptoms were also compared between groups. Differences in anorectal physiology testing were reported in patients with minimum data on anorectal manometry, endoanal ultrasonography and rectal sensory testing.

### 2.5 Data analysis

Descriptive statistics were used to show proportions of patients meeting referral diagnosis and Rome classification (graphically as Venn diagrams). Differences in risk factors, symptoms and proportions of patients with abnormal anorectal physiological measurements between patients with Rome IV defined diagnoses (faecal incontinence, functional constipation or coexistent symptoms) were compared using chi-square tests (categorical variables), parametric and non-parametric ANOVA methods (continuous variables); *p*<0·01 was considered statistically significant allowing for multiple comparisons. Similar analyses of patients referred for faecal incontinence and those reclassified were also performed. Symptom severity in the three patient groups was visualised by colour density plots using Cleveland Clinic constipation [18] and St Marks incontinence scores [19].

### 2.6 Role of funding source

There was no funding source for this study. The corresponding author had full access to the data and had final responsibility for the decision to submit for publication.

### 2.7 Ethical approval

This study was qualified as being exempt from full Research Ethical Committee review. Local sponsorship has been issued by the Queen Mary University of London (IRAS ID 270602; 18th September 2019).

## 3 Results

### 3.1 Study sample

A total of 4660 patients met the three inclusions concerning minimum dataset. Of these, 633 (13·5%) patients were excluded due to a referral other than for faecal incontinence and/or constipation (*n* = 304; 6·5%), or symptoms of faecal incontinence or functional constipation not meeting Rome IV core criteria (*n* = 329; 7·0%). This left a study sample of 4027 patients (3370 females [83·7%]). Median age was 52 years (interquartile range 41–63); 2852 females (84·6%) were parous.

### 3.2 Diagnosis by symptom questionnaires

**Fig. 1:**
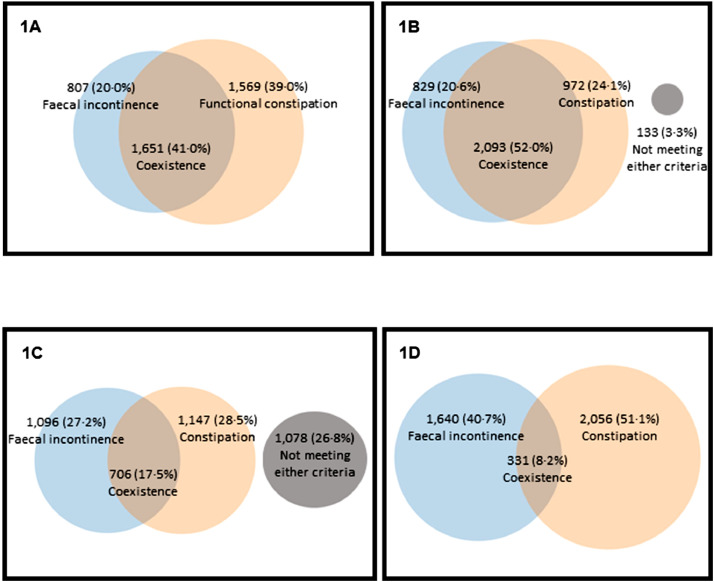
Symptoms of faecal incontinence in isolation, constipation in isolation and coexistent faecal incontinence and constipation in 4027 patients, classified by: **A.** Self-reported symptoms: Rome IV core criteria. **B.** Self-reported symptoms: St Marks incontinence score (cut-off: ≥6) and Cleveland Clinic constipation score (cut-off: ≥9). **C.** Self-reported symptoms: St Marks incontinence score (cut-off: ≥12) and Cleveland Clinic constipation score (cut-off: ≥15). **D.** Primary reason for referral stated in the clinician's referral letter.

Applying Rome IV core criteria, 807 (20·0%) patients self-reported faecal incontinence in isolation, 1569 (39·0%) patients self-reported symptoms of functional constipation in isolation, and 1651 (41·0%) self-reported coexistent faecal incontinence and functional constipation (Fig. 1A). Such overlap was replicated using St Marks incontinence (cut-off: ≥6) and Cleveland Clinic constipation scores (cut-off: ≥9) (20·6% vs 24·1% vs 52·0%, respectively: Fig. 1B); however, using these cut offs, 133 patients (3·3% of total cohort) did not meet criteria for faecal incontinence or constipation. Using more rigorous cut-offs to capture a more symptomatically severe phenotype (St Marks incontinence score: ≥12; Cleveland Clinic constipation score: ≥15), coexistent symptoms were still present in 706 (17·5%) patients (Fig. 1C).

### 3.3 Coexistent symptoms are frequently missed by referring physicians

**Fig. 2:**
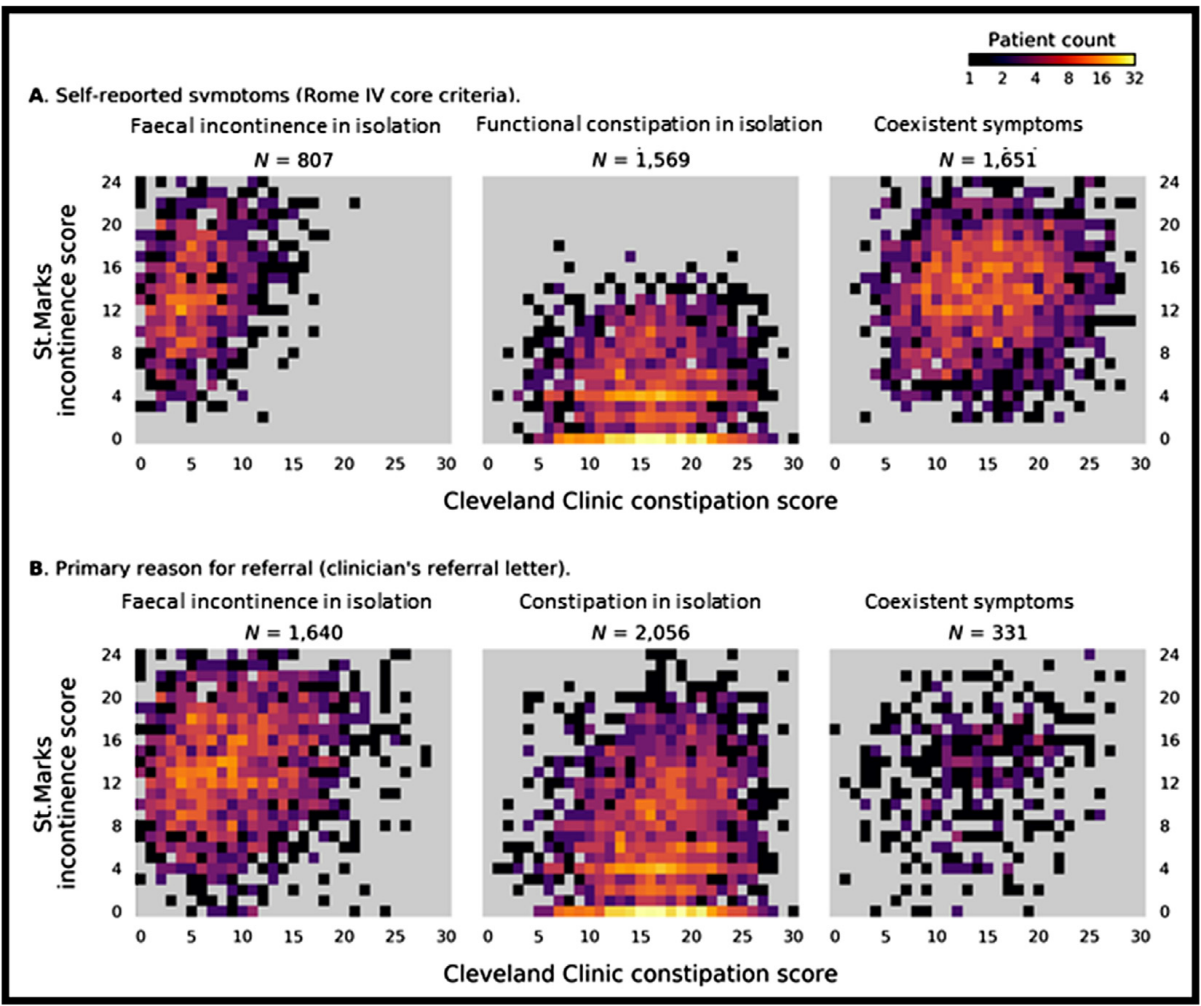
Colour density plots showing symptom severity of faecal incontinence (St Marks incontinence score: Y-axis) and constipation (Cleveland Clinic constipation score: X-axis) in 4027 patients, classified by: **A.** Self-reported symptoms: Rome IV core criteria. **B**. Primary reason for referral stated in the clinicians’ referral letter. The right panels in A and B show that coexistent faecal incontinence and constipation are frequently missed by the referring clinician; B shows that patients with all degrees of symptom severity are missed (i.e. not just those with less severe symptoms).

Scrutiny of referral letters showed that 1640 (40·7%) patients were referred for faecal incontinence in isolation, 2056 (51·1%) patients for constipation in isolation, and only 331 (8·2%) for coexistent symptoms (Fig. 1D). Colour density plots (Fig. 2a and 2b), showing the relationship between diagnosis by Rome, referring clinician and other scoring systems, clearly demonstrate that identification of coexistent symptoms did not reflect missing less severe symptoms.

**Fig. 3:**
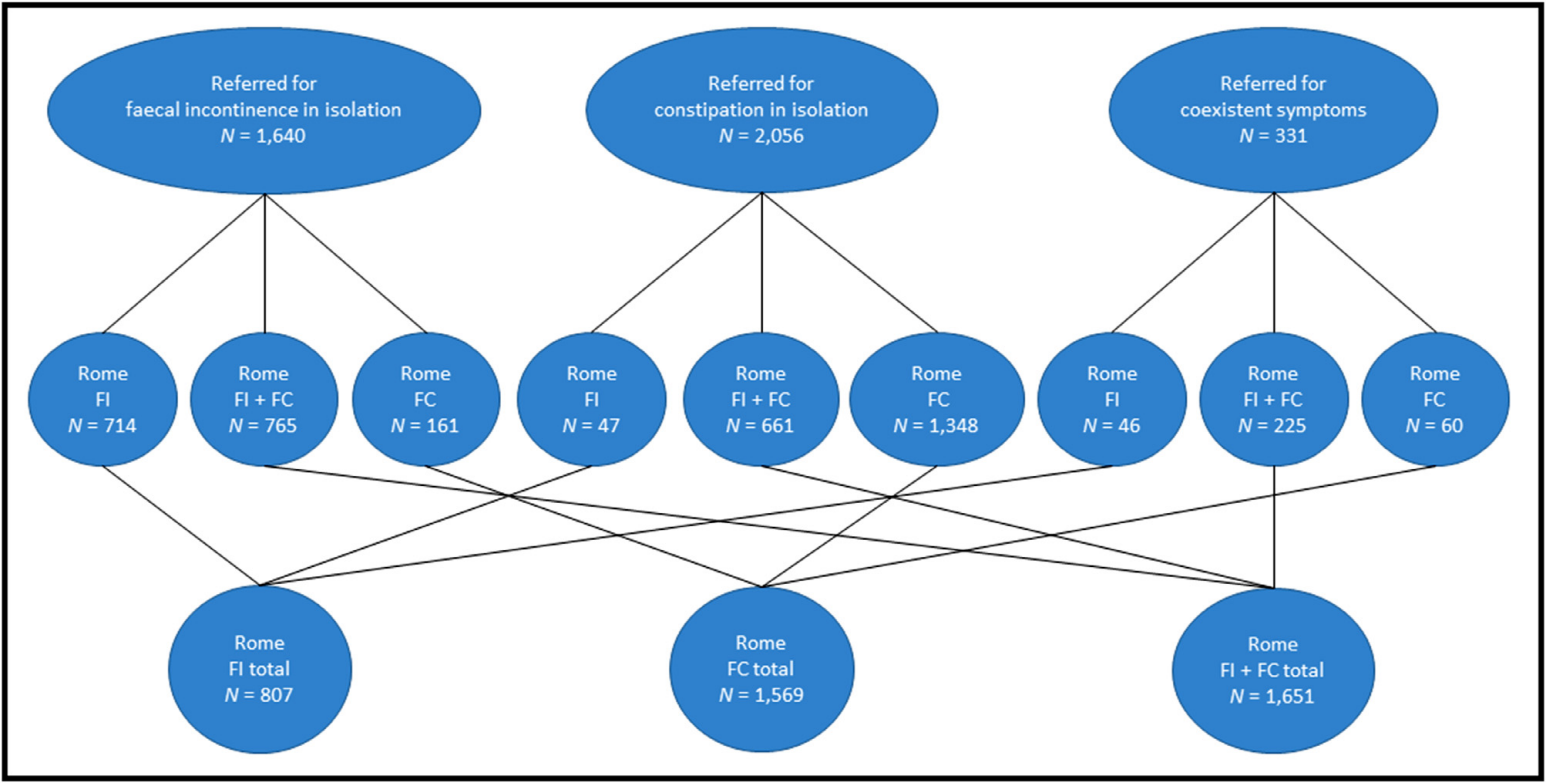
Relationships between primary reason for referral (upper three panels) and Rome reclassification according to patient-reported symptoms (middle and lower panels). FI = faecal incontinence; FC = functional constipation.

All relationships between referral symptoms and Rome IV classification are shown in Fig. 3. Coexistent symptoms were most often missed for patients referred for faecal incontinence in isolation. In this group of 1640 patients, 765 (46·7%) had concomitant functional constipation. Further, 161 patients (9·8%) referred for faecal incontinence in isolation did not meet the Rome IV criteria for faecal incontinence (episodes ≤monthly), but *did* meet those for functional constipation – an overall misclassification rate of 926/1640 (56·5%). The proportion of patients referred for faecal incontinence in isolation in whom symptoms of constipation were not mentioned by the referring clinician was constant throughout the 12-year study period (Supplementary Fig. 1). Looked at in reverse, of 1651 patients with coexistent symptoms based on Rome, only 225 (13·6%) were correctly recognised by the referrer i.e. 86·4% were missed.

### 3.4 Coexistent symptoms are reported in all age groups and in both sexes (Fig. 4)

**Fig. 4:**
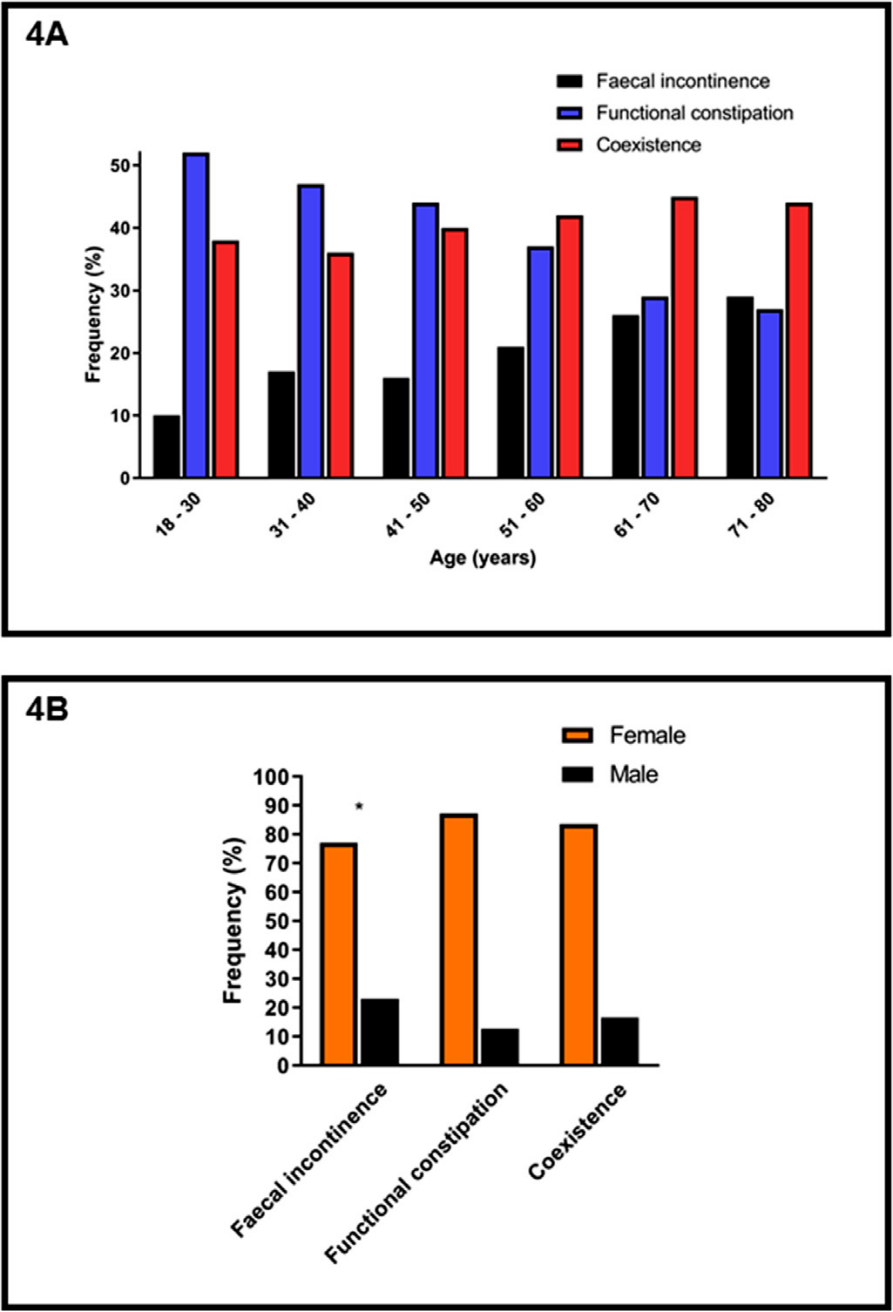
Proportions of patients with self-reported symptoms of faecal incontinence in isolation, functional constipation in isolation and coexistent faecal incontinence and functional constipation according to the Rome IV core criteria in 4027 patients: **A** Effect of age. **B.** Effect of sex. The proportion of males was higher in patients with faecal incontinence in isolation compared to patients with functional constipation in isolation or coexistent symptoms (* *p* <0·0001).

Fig. 4A shows the distribution of patients with self-reported faecal incontinence, functional constipation and coexistent symptoms (based on Rome IV core criteria) amongst different age groups. The proportion of patients with Rome diagnosis of faecal incontinence in isolation increased with increasing age (9·6% [18–30 years] to 29·1% [71–80 years]), functional constipation in isolation decreased with increasing age (52·0% [18–30 years] to 27·4% [71–80 years]), whereas coexistent symptoms remained constant across age groups (range 36·1% [18–30 years] to 44·7% [61–70 years]). Males were over-represented in the faecal incontinence in isolation group compared to other groups (Fig. 4B).

### 3.5 Putative risk factors and self-reported disease classification (Table 1)

Table 1. Patients with Rome IV diagnosis of functional constipation in isolation were significantly younger compared to patients with faecal incontinence in isolation or to those with coexistent symptoms (median age 49 vs 57 and 54 years; *p*<0·0001). All three groups had a very high proportion of parous females, and this proportion was significantly higher in patients with faecal incontinence in isolation or coexistent symptoms. A history of anal/perineal surgery was more often reported in patients with faecal incontinence in isolation compared to patients with functional constipation in isolation (23·9% vs 18·7%; *p* = 0·003). Patients with coexistent symptoms more often had a history of rectal or pelvic surgery compared to patients with faecal incontinence (rectal: 8·8% vs 3·1%; *p*<0·0001; pelvic: 39·2% vs 33·0%; *p* = 0·003). Opioid and antidepressant usage were more common in patients with coexistent symptoms compared to other groups.

**Table 1:** Risk factors in patients with symptoms of faecal incontinence in isolation, functional constipation in isolation and coexistent faecal incontinence and functional constipation, defined by the Rome IV core criteria.

	Faecal incontinence *N* = 807 (20·0%)	Functional constipation *N* = 1569 (39·0%)	Coexistent symptoms *N* = 1651 (41·0%)	Model *p* value
Age, median (IQR) Sex (%) Female Male Obstetric history (%) Nulliparous Parous Traumatic delivery^1^ Instrumental delivery^1^ Caesarean section^1^ Surgical history (%) Anal/perineal Abdominal/bowel Rectal Pelvic, including hysterectomy Comorbidities^2^ (%) Diabetes (%) Opioids (%) Antidepressants (%) Childhood bowel problems (%)	57 (46 – 66) 621 (77·0) 186 (23·0) 51 (8·2) 570 (91·8) 459 (80·5) 161 (28·2) 84 (14·7) 193 (23·9) 208 (25·8) 25 (3·1) 266 (33·0) 45 (5·6) 116/668 (17·4) 88 (10·9) 159 (19·7) 37/783 (4·7)	49 (38 – 59) 1370 (87·3) 199 (12·7) 286 (20·9) 1084 (79·1) 817 (75·4) 260 (24·0) 186 (17·2) 294 (18·7) 410 (26·1) 95 (6·1) 547 (34·9) 199 (12·7) 79/1314 (6·0) 195 (12·4) 309 (19·7) 284/1527 (18·6)	54 (42 – 64) 1379 (83·5) 272 (16·5) 181 (13·1) 1198 (86·9) 908 (75·8) 305 (25·5) 194 (16·2) 356 (21·6) 475 (28·8) 146 (8·8) 648 (39·2) 219 (13·3) 170/1269 (13·4) 285 (17·3) 424 (25·7) 231/1590 (14·5)	**< 0·0001**^a^**^,^**^b^**^,^**^c^ **-** **< 0·0001**^a^**^,^**^b^**^,^**^c^ **-** **< 0·0001**^a^**^,^**^b^**^,^**^c^ 0·044 0·168 0·446 **0·010**^a^ 0·151 **< 0·0001**^a^**^,^**^b^**^,^**^c^ **0·003**^b^ **< 0·0001**^a^**^,^**^b^ **< 0·0001**^a^**^,^**^c^ **< 0·0001**^b^**^,^**^c^ **< 0**·**0001**^b^**^,^**^c^ **< 0**·**0001**^a^**^,^**^b^**^,^**^c^

Legend:

IQR = interquartile range.

Denominators indicate variables with missing values.

Footnote:

1 Of parous females.

2 Lower back pain, fibromyalgia, chronic fatigue syndrome, headache (including migraine), joint hypermobility syndrome (≥2).

a Post hoc test: significant difference (*p* < 0·01) between faecal incontinence and functional constipation group.

b Post hoc test: significant difference (*p* < 0·01) between faecal incontinence and coexistence group.

c Post hoc test: significant difference (*p* < 0·01) between functional constipation and coexistence group.

### 3.6 Symptom profiles and self-reported disease classification (Table 2)

Table 2. Several incontinence symptoms were numerically more common in patients with Rome coexistent diagnoses compared to the faecal incontinence group (e.g. poor discrimination between stool and flatus). In contrast, urge faecal incontinence alone (i.e. vs mixed/passive symptoms) was statistically more frequent in patients with faecal incontinence in isolation (18·3% vs 11·7%: *p*<0·0001), as was frequent defaecation (58·1% vs 35·8%; *p*<0·0001).

**Table 2:** Symptoms in patients with faecal incontinence in isolation, functional constipation in isolation and coexistent faecal incontinence and constipation, defined by the Rome IV core criteria.

	Faecal incontinence *N* = 807 (20·0%)	Functional constipation *N* = 1569 (39·0%)	Coexistent symptoms *N* = 1651 (41·0%)	Model *p* value
**Faecal incontinence** (%)				
Combined solid and liquid stool Duration of symptoms (> 5 years) Type Urge Passive Post-defaecation Cough Mixed Flatus incontinence Frequency > monthly Duration of symptoms (> 5 years) Pads/plugs Constipation medication (e.g. Loperamide) Urgency Poor discrimination between stool and flatus	242/804 (30·1) 144/786 (18·3) 71/786 (9·0) 69/786 (8·5) 11/786 (1·4) 491/786 (62·4) 532 (65·9) 161/501 (32·1) 426 (52·8) 192 (23·8) 643 (79·7) 384/787 (48·8)	- - - - - - 707 (45·1) 272/662 (41·1) 133 (8·5) 234 (14·9) 580 (37·0) 326/1508 (21·6)	613/1620 (37·8) 182/1556 (11·7) 138/1556 (8·9) 155/1556 (10·0) 34/1556 (2·2) 1047/1556 (67·3) 1299 (78·7) 531/1204 (44·1) 866 (52·5) 404 (24·5) 1196 (72·4) 944/1588 (59·4)	**0·0002** **< 0·0001** 0·895 0·358 0·191 0·020 **< 0·0001**^a^**^,^**^b^**^,^**^c^ **< 0·0001**^a^**^,^**^b^ **< 0·0001**^a^**^,^**^c^ **< 0·0001**^a^**^,^**^c^ **< 0·0001**^a^**^,^**^b^**^,^**^c^ **< 0·0001**^a^**^,^**^b^**^,^**^c^
**Constipation** (%)				
Duration of symptoms (>10 years) Time on lavatory (>10 min) Oral laxative use Unsuccessful bowel movements (>25%) Painful defaecation (>25%) Abdominal pain (>25%) Bloating (>25%) **Straining (≥25%)** **Incomplete rectal emptying (≥25%)** **Anorectal blockage (≥25%)** **Manual manoeuvres (≥25%)**	- 71 (8·8) 62/771 (8·0) 48 (5·9) 200 (24·8) 357 (44·2) 145/787 (18·4) 61 (7·6) 416 (51·5) 9 (1·1) 5 (0·6)	670 (42·7) 766 (48·8) 902/1479 (61·0) 1141 (72·7) 1089 (69·4) 1115 (71·1) 607/1548 (39·2) 1428 (91·0) 1496 (95·3) 1241 (79·1) 738 (47·0)	592 (35·9) 739 (44·8) 729/1537 (47·4) 1084 (65·7) 1080 (61·2) 1160 (70·3) 665/1616 (41·2) 1416 (85·8) 1580 (95·7) 1217 (73·7) 625 (37·9)	**< 0·0001** **< 0·0001**^a^**^,^**^b^ **< 0·0001**^a^**^,^**^b^**^,^**^c^ **< 0·0001**^a^**^,^**^b^**^,^**^c^ **< 0·0001**^a^**^,^**^b^ **< 0·0001**^a^**^,^**^b^ **< 0·0001**^a^**^,^**^b^ **< 0·0001**^a^**^,^**^b^**^,^**^c^ **< 0·0001**^a^**^,^**^b^ **< 0·0001**^a^**^,^**^b^**^,^**^c^ **< 0·0001**^a^**^,^**^b^**^,^**^c^
**Prolapse** (%)				
Feeling of bulge Blood loss per rectum Mucous discharge per rectum	209/784 (26·7) 235/771 (30·5) 407/771 (52·8)	746/1522 (49·0) 709/1515 (46·8) 809/1503 (52·8)	942/1604 (58·7) 810/1589 (51·0) 1096/1565 (70·0)	**< 0·0001**^a^**^,^**^b^**^,^**^c^ **< 0·0001**^a^**^,^**^b^ **< 0·0001**^b^**^,^**^c^
**Other** (%)				
Bowel frequency **Infrequent (≤3x per week)** Normal (1 – 2x per 1 – 2 days) Frequent (≥3x per day) Stool consistency **Hard (Bristol 1 – 2)** Normal (Bristol 3 – 5) Liquid (Bristol 6 – 7) Variable IBS (Rome III criteria)	10/792 (1·3) 322/792 (40·7) 460/792 (58·1) 4/795 (0·5) 254/795 (31·9) 180/795 (22·6) 357/795 (44·9) 78/408 (19·1)	667/1521 (43·9) 552/1521 (36·3) 302/1521 (19·9) 443/1556 (28·5) 189/1556 (12·1) 89/1556 (5·7) 835/1556 (53·7) 324/891 (36·4)	501/1589 (31·5) 519/1589 (32·7) 569/1589 (35·8) 281/1639 (17·1) 251/1639 (15·3) 163/1639 (10·0) 944/1639 (57·6) 359/894 (40·2)	**< 0·0001**^a^**^,^**^b^**^,^**^c^ **0·0005**^b^ **< 0·0001**^a^**^,^**^b^**^,^**^c^ **< 0·0001**^a^**^,^**^b^**^,^**^c^ **< 0·0001**^a^**^,^**^b^**^,^**^c^ **< 0·0001**^a^**^,^**^b^**^,^**^c^ **< 0·0001**^a^**^,^**^b^ **< 0·0001**^a^**^,^**^b^

Legend:

Underlined symptoms are part of the Rome IV diagnostic criteria for functional constipation[1].

Denominators indicate variables with missing values.

a Post hoc test: significant difference (*p* < 0·01) between faecal incontinence and functional constipation group.

b Post hoc test: significant difference (*p* < 0·01) between faecal incontinence and coexistence group.

c Post hoc test: significant difference (*p* < 0·01) between functional constipation and coexistence group.

Symptoms of constipation were present in all groups including the faecal incontinence group (e.g. incomplete rectal emptying in 51·5% patients). Between the functional constipation and coexistent groups, symptom burden was almost always higher in the former with notable examples being oral laxative use (61·0% vs 47·4%) and manual manoeuvres to facilitate defaecation (47·0% vs 37·9%). Clearly, symptoms required to make a Rome classification of functional constipation were dramatically higher in these two groups than in patients with faecal incontinence (this being a *fait accompli* of the criteria). Prolapse symptoms (feeling of bulge, blood loss per rectum, mucous discharge per rectum) were prevalent in all groups but greatest in those with coexistent Rome diagnoses.

To acknowledge the importance of loose stools as a risk factor for faecal incontinence we performed further subgroup analyses. Supplementary Fig. 2A and 2B show the distribution of symptoms after excluding 346 patients with predominantly loose stools not taking oral laxatives. The proportion of patients with coexistent symptoms remained unchanged. Similarly, symptom presentation in this subgroup also remained unchanged (Supplementary Table 1). In those patients with loose stools not taking oral laxatives (Supplementary Fig. 2C and D), symptoms of faecal incontinence in isolation were frequently reported. However, coexistent symptoms were still present in this group (31·8% based on Rome IV core criteria; 47·4% based on Cleveland Clinic constipation and St Marks incontinence scores).

### 3.7 Anorectal physiological measurements and self-reported disease classification (Table 3)

#### 3.7.1 Anal sphincter structure and function

Table 3 Using endoanal ultrasonography, patients with faecal incontinence alone were more likely to have disrupted anal sphincters compared to patients with functional constipation or those with coexistent diagnoses. In contrast, anal sphincter dysfunction (based on manometry) was found frequently in patients with faecal incontinence alone and those with coexistent symptoms (57·2% and 55·2% respectively; compared to 33·1% in the functional constipation group; *p*<0·0001). Ultrasound revealed that these two groups also differed from the functional constipation group in proportion of patients with degenerate/atrophic anal sphincters.

**Table 3:** Proportions of patients with abnormal findings on anorectal physiological testing in patients with faecal incontinence in isolation, functional constipation in isolation and coexistent faecal incontinence and functional constipation, defined by the Rome IV core criteria, in those with a minimum of anorectal manometry, rectal sensation testing and endoanal ultrasonography *(n* = 3697; 91·9% of the total study sample).

	Faecal incontinence *N* = 750 (20·3%)	Functional constipation *N* = 1438 (38·9%)	Coexistent symptoms *N* = 1509 (40·8%)	P-value
Anorectal manometry^1^ (%) Normal Anal hypotension + normal contractility Anal normotension + hypocontractility Anal hypotension + hypocontractility	321 (42·8) 86 (11·5) 183 (24·4) 160 (21·3)	962 (66·9) 88 (6·1) 296 (20·6) 92 (6·4)	676 (44·8) 177 (11·7) 385 (25·5) 271 (18·0)	**< 0·0001**^a^**^,^**^c^ **< 0·0001**^a^**^,^**^c^ **0·005**^c^ **< 0·0001**^a^**^,^**^c^
Endoanal ultrasonography (%) *Internal anal sphincter* Intact Degenerate/atrophic Disrupted Abnormal, focal *External anal sphincter* Intact Degenerate/atrophic Disrupted Abnormal, focal	382 (50·9) 187 (24·9) 170 (22·7) 49 (6·5) 345 (46·0) 65 (8·7) 272 (36·3) 98 (13·1)	1114 (77·5) 173 (12·0) 104 (7·2) 74 (5·1) 945 (65·7) 57 (4·0) 257 (17·9) 198 (13·8)	892 (59·1) 323 (21·4) 267 (17·6) 94 (6·2) 774 (51·3) 139 (9·2) 456 (30·2) 201 (13·3)	**< 0·0001**^a^**^,^**^b^**^,^**^c^ **< 0·0001**^a^**^,^**^c^ **< 0·0001**^a^**^,^**^b^**^,^**^c^ 0·314 **< 0·0001**^a^**^,^**^c^ **< 0·0001**^a^**^,^**^c^ **< 0·0001**^a^**^,^**^b^**^,^**^c^ 0·886
Rectal sensation to balloon distension^1^ (%) Normal Rectal hypersensitivity Rectal hyposensitivity	620 (82·7) 61 (8·1) 69 (9·2)	1164 (80·9) 57 (4·0) 217 (15·1)	1252 (83·0) 72 (4·8) 185 (12·3)	0·326 **0·0001**^a^**^,^**^b^ **0·0004**^a^
Whole-gut transit studies (%) Delayed	81 (10·8) 17 (21·0)	851 (59·2) 376 (44·2)	510 (33·8) 165 (32·4)	**-**d **-**d
Defaecography (%) Functional abnormality Significant structural abnormality Intussusception Obstructing recto-rectal Recto-anal Rectocoele Depth ≥4 cm Depth 2–4 cm, symptomatic Enterocoele Megarectum Rectal prolapse Functional + structural abnormality	616 (82·1) 138 (22·4) 230 (37·3) 78 (12·7) 110 (17·9) 60 (9·7) 5 (0·8) 12 (1·9) 29/202 (14·4) 10 (1·6) 29 (4·7)	1378 (95·8) 380 (27·6) 856 (62·1) 175 (12·7) 274 (19·9) 285 (20·7) 259 (18·8) 132 (9·6) 125/677 (18·5) 16 (1·2) 168 (12·2)	1392 (92·2) 338 (24·2) 743 (53·4) 153 (11·0) 243 (17·5) 199 (14·3) 178 (12·8) 87 (6·3) 87/626 (13·9) 56 (4·0) 131 (9·4)	**< 0·0001**^a^**^,^**^b^**^,^**^c^ 0·026 **< 0·0001**^a^**^,^**^b^**^,^**^c^ 0·327 0·232 **< 0·0001**^a^**^,^**^b^**^,^**^c^ **< 0·0001**^a^**^,^**^b^**^,^**^c^ **< 0·0001**^a^**^,^**^b^**^,^**^c^ 0·063 **< 0·0001**^b^**^,^**^c^ **< 0·0001**^a^**^,^**^b^

Legend:

1 Diagnostic classification based on the London classification for disorders of anorectal function.

a Post hoc test: significant difference (*p* < 0·01) between faecal incontinence and functional constipation group.

b Post hoc test: significant difference (*p* < 0·01) between faecal incontinence and coexistence group.

c Post hoc test: significant difference (*p* < 0·01) between functional constipation and coexistence group.

d Analysis not performed due to evident selection bias.

#### 3.7.2 Rectal sensory testing

Patients with faecal incontinence were more likely to be diagnosed with rectal hypersensitivity compared to other groups (8·1% [incontinence] vs 4·0% and 4·8%; model *p*<0·0001). By contrast, rectal hyposensitivity was most frequently diagnosed in patients with functional constipation (15·1% [functional constipation] vs 9·2% and 12·3%; model *p* = 0·0004).

#### 3.7.3 Whole-gut transit time

Descriptively, data showed that 44·2% of the functional constipation group had delayed transit. Other groups had only selected application of this test and due to selection bias, these data have not been compared statistically.

#### 3.7.4 Defaecography

Excepting the caveat that slightly fewer patients in the faecal incontinence group underwent this test, defaecography revealed an isolated functional abnormality in similar proportions of patients in all groups. In respect of structure, proportions of patients with rectal intussusception were also comparable between all three groups (circa 30%). In contrast, large and smaller, symptomatic rectocoeles were most common in patients with functional constipation (together 39·5%), and higher in patients with coexistent symptoms (27·1%) compared to patients with faecal incontinence in isolation (10·5%; model *p*<0·0001). Enterocoeles were also more common in patients with functional constipation or coexistent symptoms compared to patients with faecal incontinence (9·6% and 6·3% vs 1·9%, respectively; model *p*<0·0001). Patients with coexistent diagnoses were more often found to have an overt rectal prolapse compared to patients with isolated symptoms of faecal incontinence or functional constipation.

### 3.8 Factors associated with reclassification of patients referred with faecal incontinence (Supplementary Table 2)

As noted above, of 1640 patients referred for faecal incontinence in isolation, 765 (46·7%) patients also met Rome IV core criteria for functional constipation and 161 (9·8%) were only classified as functional constipation i.e. they did not meet Rome IV criteria for incontinence (Fig. 3). On this basis, 926 (56·5%) patients could be considered ‘reclassified’. These reclassified patients did not differ in putative risk factors to those ‘remaining’ with a faecal incontinence diagnosis, with the exception of comorbidities (9·8% vs 5·2%; *p* = 0·0005), opioid use (14·5% vs 9·9%; *p* = 0·006) and childhood bowel problems (11·5% vs 4·2%; *p*<0·0001) (Supplementary Table 2A). Patients with faecal incontinence were twice as likely to have urge incontinence alone (19·0% vs 9·7%; *p*<0·0001) but mixed incontinence was significantly more common in reclassified patients (72·5% vs 62·1%; *p*<0·0001) (Supplementary Table 2B). Not surprisingly, nearly all constipation symptoms were significantly more common at the *p*<0·0001 level in reclassified patients accepting that some of these (e.g. straining, incomplete rectal emptying) were themselves criteria for group allocation. Prolapse symptoms were also more frequent in the reclassified group, especially a sensation of bulging into the vagina (50·3% vs 25·7%; *p*<0·0001).

There were no differences in anal sphincter structure or function between faecal incontinence patients and those reclassified (Supplementary Table 2C). In contrast, significant structural abnormalities, notably in respect of pelvic organ prolapse (e.g. rectocoele, enterocoele and rectal prolapse), were more common in reclassified patients.

## 4 Discussion

**Fig. 5:**
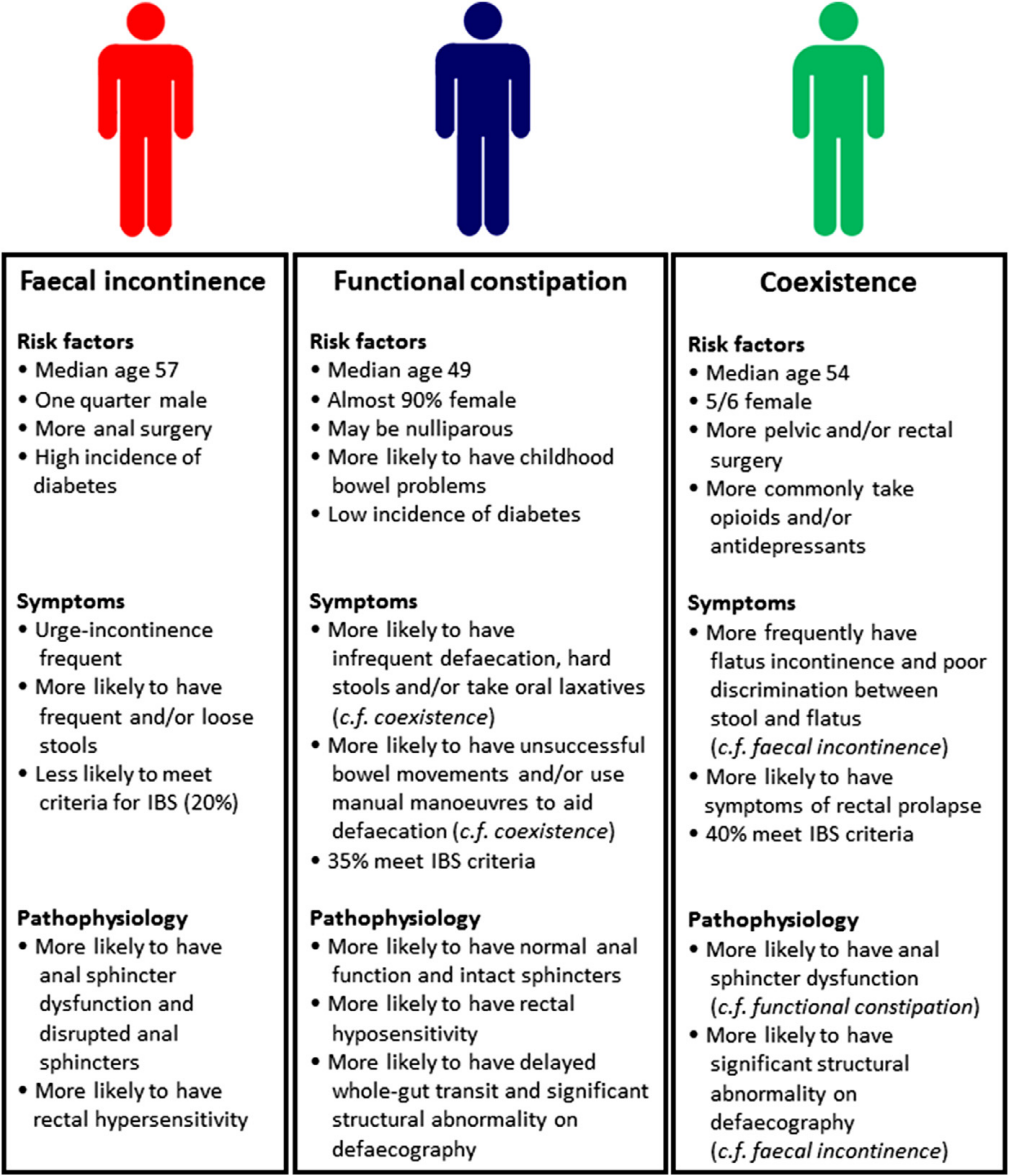
Broad phenotypic characteristics of patients in the 3 groups.

In 4027 adults referred for specialist investigation of faecal incontinence and/or constipation, we found that coexistent symptoms were self-reported in 41·0% patients using established international diagnostic (Rome) criteria [1,4]. This overlap was missed by the referrer in the majority of patients (86·4%) and broadly unchanged even after removing the possible confounding effect of loose stools on faecal incontinence symptoms. Considering those referred specifically for investigation of faecal incontinence, 46·6% had coexistent diagnoses, and almost 10% *only* met criteria for functional constipation. This is the largest study to date to recognise the magnitude of coexistent symptoms in a referral population of community-dwelling adults. Further, we have sought to explain our observations by providing comprehensive information on clinical characteristics (summarised Fig. 5).

Our results accord with observations from previous studies in specialist colorectal units that report similar evidence of concurrent symptoms [20]. Notably, a recent study in 946 patients referred for investigation of faecal incontinence showed that the majority also reported constipation symptoms (69·3% *c.f.* 61·4% in current study)[21]. The absence of awareness of this coexistence amongst referring clinicians was not evaluated in previous studies.

While emphasising that generalisability of our results to broader populations is limited by the study sample from a single UK specialist centre, our estimate of 41·0% patients with coexisting symptoms *does* closely match estimates from the few population studies where data on coexistent symptoms have been presented. For instance, in a recent population sample (focussed on faecal incontinence) of >70,000 American individuals, 37·8% of those reporting incontinence during the past week had concurrent constipation [2]. This proportion was slightly lower than a subsample of a Dutch population (44·0%)[22].

A pertinent question is why do referrers so frequently fail to recognise (or at least document in their referrals) the coexistence of symptoms when this appears to be so common? This observation, which was unchanged across 12 years in the current study, might relate to time constraints during consultation. It is well known that patients may be reticent to volunteer some bowel symptoms for fear of embarrassment [23] and systematic questioning is thus required. However, this could equally reflect misconceptions about the relevance of such questioning in different patient populations. Most clinicians will be aware of the coexistence of such symptoms in paediatric and geriatric populations [6], [7], [8], [9], [10], [11], [12]. However in adults, such questioning may be guided by a notion of risk. Here, our data show that many factors considered classical risks for incontinence were also present in patients with coexistent diagnoses e.g. advancing parity was common in all groups (79·1% to 91·8%). In fact, considering patients referred for incontinence symptoms alone, neither parity nor traumatic/instrumental delivery affected likelihood of reclassification. The same was true of anal/perineal surgery.

These observations in regard to putative risk concur with the evolving understanding of pathophysiology of defaecation disorders that moves away from a symptom-based dichotomy (trichotomy with pelvic organ prolapse) to one where it is accepted that most patients’ symptoms represent the end product of multiple risk factors and their functional/structural consequences on the colon, anorectum and pelvic floor. This is exemplified by the faecal incontinence literature. Pelvic floor denervation, secondary to constipation was historically considered one of the primary pathological mechanisms causing faecal incontinence [24]. Since the advent of endoanal ultrasonography in the 1990s, a shift in focus was observed to obstetric-related sphincter injury [25]. However, population-based studies report a similar prevalence of faecal incontinence in females and males, suggesting that the impact of obstetric-related sphincter injury has been over-estimated [2]. A recent Swedish national register-based study showed that Caesarean section as well as vaginal delivery conferred a risk for anal incontinence [26], indicating that other pregnancy-related factors than those leading to direct sphincter damage are important. Indeed, stretched and damaged ligaments can disrupt the ability of pelvic floor muscles to develop normal tension, hindering anorectal closure, as well as causing laxity and prolapse [27]. These pathophysiological concepts have also been described in constipation, secondary to chronic straining [24].

A further question is does lack of recognition of coexistent symptoms matter to clinical care? Currently, it is not uncommon for choice of referral to a gastroenterologist, colorectal surgeon or urogynaecologist to be based on a quick appraisal of main presenting symptoms (constipation, faecal incontinence, organ prolapse respectively). Getting this right probably matters. For example, to ignore coexistent constipation in a patient with faecal incontinence may result in missing beneficial expertise in biofeedback therapy [28]. Surgical augmentation of the anal sphincter with the intent of treating incontinence in the presence of obstructed defaecation may make symptoms worse not better [29]. Where suppositories may prevent passive and / or post-defaecation faecal incontinence by improving emptying of the rectum, prescription of oral laxatives in patients with coexistent symptoms may require monitoring, as faecal incontinence can be exacerbated due to resultant looser stools or diarrhoea [30,31]

Similarly, to miss a patient with significant pelvic organ prolapse may lead to futile attempts at symptom control when they might better have been directed early to pelvic floor muscle training +/- surgical repair. In our study, prolapse symptoms, especially a sensation of bulge into the vagina, aligned closely with a change in classification. This finding was borne out by radiology testing, where structural abnormalities (e.g. rectocoele, enterocoele, rectal prolapse) were more commonly observed in patients with coexistent symptoms. There are reasonable published data to suggest that surgical rectocoele correction may improve all main defaecatory symptoms (including incontinence and constipation) [32].

Interestingly, this did not hold for internal rectal prolapse where significant degrees (i.e. recto-anal) were equally common in all groups. This has relevance to surgical management, where it has been assumed that anatomical correction of high grade intussusception is mainly predicated on treating constipation secondary to obstructed defaecation [33]. In fact, there are data showing significant benefits for patients with mainly incontinence symptoms with the suggestion that such surgical correction should precede interventions primarily directed at restoration of sphincter function [34].

In drawing conclusions, we acknowledge certain limitations. The first concerns sampling. Our population was drawn from the referral practice of a single large UK centre. While our results concur with others (see earlier discussion), there was undoubtedly a skew toward patients referred for surgical care (the GI Physiology Unit being primarily led by surgeons). Secondly, we acknowledge that our design is cross-sectional. We simply cannot comment whether the subsequent course of patients was influenced favourably by our recognition of coexistent symptoms, and we have been careful to report on associations and not on causation. It is clear however that our practice takes heed of coexistent symptoms e.g. proctography was employed in >90% patients with coexistent symptoms. All patients with significant structural abnormalities are thence reviewed at a pelvic floor MDT if surgery is considered. This accords with national UK guidance, and it is now recognised that dedicated pelvic floor units are required in order to provide high quality care to patients with pelvic floor disorders [35]. A final criticism is that our reclassification is based on Rome criteria that may themselves be criticised as a final arbiter of diagnosis [36]. In defence, the same or greater overlap was replicated using other well-accepted scoring systems [18,19].

Accepting these caveats, we conclude that over 40% of patients referred for anorectal physiological investigation had coexistent faecal incontinence and functional constipation, based on validated criteria (Rome or otherwise). This overlap was not acknowledged by the referrer for 86·4% patients. Lack of recognition of coexistence of faecal incontinence and constipation is at the very least an educational issue and one that may have important treatment and research implications (in the era of stratified medicine). If a further iteration of Rome is undertaken, our findings could invite recognition of this overlap in the manner that Rome IV has done for IBS-C and functional constipation [1].

## Author contributions

S Mark Scott and Charles H Knowles conceived the study design. Paul F Vollebregt and S Mark Scott collected the study data. All authors analysed the data. Paul F Vollebregt, Charles H Knowles and S Mark Scott wrote the manuscript. All authors edited the manuscript and approved the final version.

## Funding

None.

## Data sharing agreement

The data that support the findings of this study are available from the corresponding author upon reasonable request.

## Declaration of Competing Interest

Paul F Vollebregt, Lukasz Wiklendt and Phil G Dinning have no conflict of interest. Charles H Knowles has received financial remuneration from Medtronic Inc. as speaker fees and for expert advisory committees, and research support from Saluda Medical. S Mark Scott has received honoraria for teaching from MMS/Laborie.

## Appendix Supplementary materials

Image, application 1

Image, application 2
