# Supplementary material for: Coexistent faecal incontinence and constipation: A cross-sectional study of 4027 adults undergoing specialist assessment
Source: eClinicalMedicine. 2020 Oct 13;27:100572. doi: 10.1016/j.eclinm.2020.100572 (PMC7599308; doi:10.1016/j.eclinm.2020.100572)
Supplement: Supplementary file 1 [file mmc1.docx]

**SUPPLEMENTARY DOCUMENT**

**Supplementary Figure 1. Proportions of patients referred for faecal incontinence in isolation, in whom self-reported symptoms of functional constipation were not mentioned by the referrer.**

**Legend:**

Year of referral was missing in 2 (0·1%) of the 1,640 patients referred for faecal incontinence in isolation within the 12-year study period.

**Supplementary Figure 2. Symptoms of faecal incontinence in isolation, constipation in isolation and coexistent faecal incontinence and constipation, classified by Rome IV core criteria (panel A + C), or by St Marks incontinence score (cut-off: ≥6) and Cleveland Clinic constipation score (cut-off: ≥9) (Panel B + D):**

**3,681 patients (excluding 346 patients with predominantly loose stools who were not on oral laxatives).**


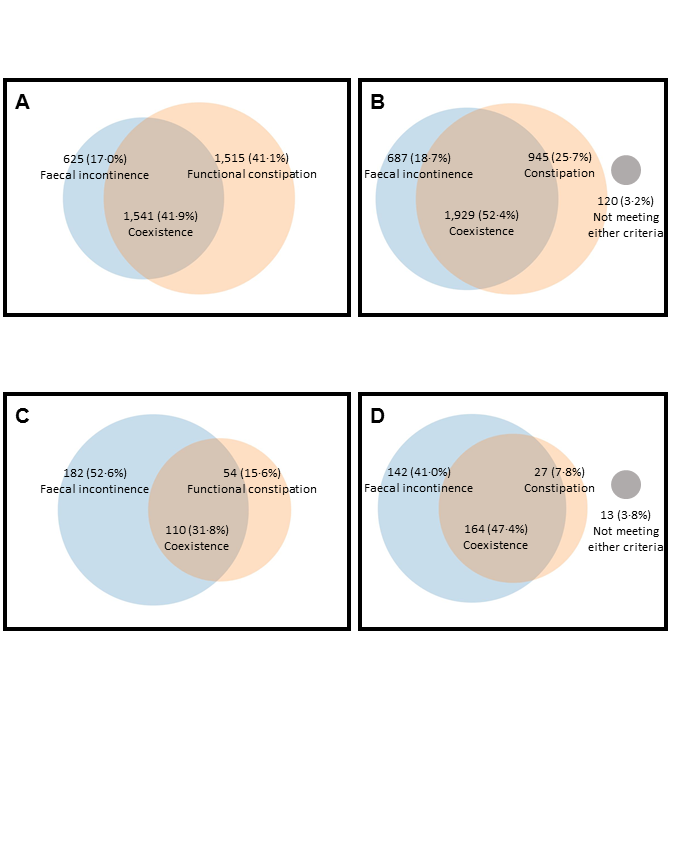


346 patients with predominantly loose stools and not taking oral laxatives.


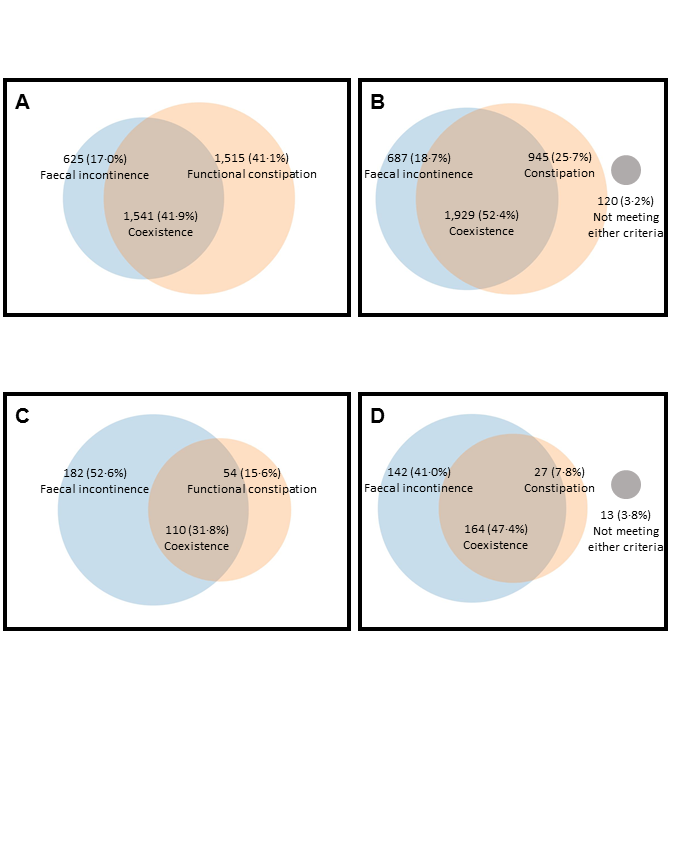


**Supplementary Table 1. Symptoms of faecal incontinence in isolation, functional constipation in isolation and coexistent faecal incontinence and constipation, defined by the Rome IV core criteria, in 3,681 patients (excluding 346 patients with predominantly loose stools who were not on oral laxatives).**

|  | Faecal incontinence  *N* = 625 (17·0%) | Functional constipation  *N* = 1,515 (41·1%) | Coexistent symptoms  *N* = 1,541 (41·9%) | Model *p* value |
| --- | --- | --- | --- | --- |
| **Faecal incontinence** (%) | | | |  |
| Combined solid and liquid stool  Duration of symptoms (> 5 years)  Type  Urge  Passive  Post-defaecation  Cough  Mixed  Flatus incontinence  Frequency > monthly  Duration of symptoms (> 5 years)  Pads/plugs  Constipation medication (e.g. Loperamide)  Urgency  Poor discrimination between stool and flatus | 181/623 (29·1)  102/609 (16·8)  52/609 (8·5)  62/609 (10·2)  9/609 (1·5)  384/609 (63·1)  404 (64·6)  121/378 (32·0)  323 (51·7)  130 (20·8)  485 (77·6)  276/609 (45·3) | -  -  -  -  -  -  687 (45·4)  264/643 (41·1)  127 (8·4)  228 (15·1)  554 (36·6)  314/1,456 (21·6) | 569/1,512 (37·6)  169/1,451 (11·7)  130/1,45 (9·0)  150/1,451 (10·3)  33/1,451 (2·3)  969/1,451 (66·8)  1,207 (78·3)  493/1,123 (43·9)  807 (52·4)  375 (24·3)  1,104 (71·6)  878/1,480 (59·3) | **0·0002**  **0·002**  0·759  0·915  0·243  0·104  **< 0·0001 ^a, b, c^**  **< 0·0001 ^a, b^**  **< 0·0001 ^a, c^**  **< 0·0001 ^a, c^**  **< 0·0001 ^a, b, c^**  **< 0·0001 ^a, b, c^** |
| **Constipation** (%) | | | |  |
| Duration of symptoms (>10 years)  Time on lavatory (>10 minutes)  Oral laxative use  Unsuccessful bowel movements (>25%)  Painful defaecation (>25%)  Abdominal pain (>25%)  Bloating (>25%)  **Straining (>25%)**  **Incomplete rectal emptying (>25%)**  **Anorectal blockage (>25%)**  **Manual manoeuvres (>25%)** | -  53 (8·5)  62/602 (10·3)  37 (5·9)  132 (21·1)  244 (39·0)  100/610 (16·4)  49 (7·8)  320 (51·2)  7 (1·1)  4 (0·6) | 658 (43·4)  736 (48·6)  902/1,429 (63·1)  1,113 (73·5)  1,063 (70·2)  1,080 (71·3)  589/1,494 (39·4)  1,387 (91·6)  1,443 (95·3)  1,207 (79·7)  726 (47·9) | 575 (37·3)  703 (45·6)  729/1,442 (50·6)  1,034 (67·1)  1,019 (66·1)  1,078 (70·0)  614/1,508 (40·7)  1,342 (87·1)  1,474 (95·7)  1,150 (74·6)  593 (38·5) | **0·0006**  **< 0·0001 ^a, b^**  **< 0·0001 ^a, b, c^**  **< 0·0001 ^a, b, c^**  **< 0·0001 ^a, b^**  **< 0·0001 ^a, b^**  **< 0·0001 ^a, b^**  **< 0·0001 ^a, b, c^**  **< 0·0001 ^a, b^**  **< 0·0001 ^a, b, c^**  **< 0·0001 ^a, b, c^** |
| **Prolapse** (%) | | | |  |
| Feeling of bulge  Blood loss per rectum  Mucous discharge per rectum | 160/610 (26·2)  165/597 (27·6)  300/596 (50·3) | 723/1,469 (49·2)  689/1,464 (47·1)  780/1,455 (53·6) | 888/1,500 (59·2)  753/1,480 (50·9)  1,025/1,459 (70·3) | **< 0·0001 ^a, b, c^**  **< 0·0001 ^a, b^**  **< 0·0001 ^b, c^** |
| **Other** (%) | | | |  |
| Bowel frequency  **Infrequent (<3x per week)**  Normal (1 – 2x per 1 – 2 days)  Frequent (≥3x per day)  Stool consistency  **Hard (Bristol 1 – 2)**  Normal (Bristol 3 – 5)  Liquid (Bristol 6 – 7)  Variable  IBS (Rome III criteria) | 8/613 (1·3)  270/613 (44·1)  335/613 (54·7)  4 (0·6)  254 (40·6)  10 (1·6)  357 (57·1)  53/314 (16·9) | 693/1,503 (46·1)  531/1,503 (35·3)  279/1,503 (18·6)  444/1,509 (29·4)  189/1,509 (12·5)  41/1,509 (2·7)  835/1,509 (55·3)  317/859 (36·9) | 527/1,523 (34·6)  494/1,523 (32·4)  502/1,523 (33·0)  282/1,536 (18·4)  251/1,536 (16·3)  59/1,536 (3·8)  944/1,536 (61·5)  334/835 (40·0) | **< 0·0001 ^a, b, c^**  **< 0·0001 ^a, b^**  **< 0·0001 ^a, b, c^**  **< 0·0001 ^a, b, c^**  **< 0·0001 ^a, b, c^**  0.015  **0·002 ^c^**  **< 0·0001 ^a, b^** |

**Legend:**

Underlined symptoms are part of the Rome IV diagnostic criteria for functional constipation^1^

^a^ Post hoc test: significant difference (*p* < 0·01) between faecal incontinence and functional constipation group

^b^ Post hoc test: significant difference (*p* < 0·01) between faecal incontinence and coexistence group

^c^ Post hoc test: significant difference (*p* < 0·01) between functional constipation and coexistence group

Denominators indicate variables with missing values

**Supplementary Table 2A. Risk factors in patients referred for symptoms of faecal incontinence in isolation, reclassified according to Rome IV core criteria.**

|  | Referred for faecal incontinence in isolation  *N* = 1,640 | Reclassification according to Rome IV core criteria | | | | *p* value^b^ |
| --- | --- | --- | --- | --- | --- | --- |
|  |  | Faecal incontinence  *N* = 714 (43·5%) | Coexistent symptoms  *N* = 765 (46·6%) | Functional constipation  *N* = 161 (9·8%) | Reclassified^a^  *N* = 926 (56·5%) |  |
| Age, median (IQR)  Sex (%)  Female  Male  Obstetric history (%)  Nulliparous  Parous  Traumatic delivery^1^  Instrumental delivery^1^  Caesarean section^1^  Surgical history (%)  Anal/perineal  Abdominal/bowel  Rectal  Pelvic, including hysterectomy  Comorbidities^2^ (%)  Diabetes (%)  Opioids (%)  Antidepressants (%)  Childhood bowel problems (%) | 57 (45 – 66)  1,291 (78·7)  349 (21·3)  111 (8·6)  1,180 (91·4)  953 (80·8)  344 (29·2)  179 (15·2)  380 (23·2)  433 (26·4)  67 (4·1)  538 (32·8)  128 (7·8)  198/1,311 (15·1)  205 (12·5)  353 (21·5)  131/1,584 (8·3) | 57 (46 – 67)  549 (76·9)  165 (23·1)  44 (8·0)  505 (92·0)  414 (82·0)  149 (29·6)  74 (14·7)  166 (23·2)  183 (25·6)  20 (2·8)  230 (32·2)  37 (5·2)  103/589 (17·5)  71 (9·9)  133 (18·6)  29/694 (4·2) | 58 (45 – 66)  609 (79·6)  156 (20·4)  51 (8·4)  558 (91·6)  445 (79·7)  159 (28·5)  91 (16·3)  178 (23·3)  208 (27·2)  44 (5·8)  267 (34·9)  75 (9·8)  82/585 (14·0)  120 (15·7)  191 (25·0)  80/731 (10·9) | 50 (35 – 62)  133 (82·6)  28 (17·4)  16 (12·0)  117 (88·0)  94 (80·3)  36 (30·8)  14 (12·0)  36 (22·4)  42 (26·1)  3 (1·9)  41 (25·5)  16 (9·9)  13/137 (9·5)  14 (8·7)  29 (18·0)  22/159 (13·8) | 56 (44 – 66)  742 (80·1)  184 (19·9)  67 (9·0)  675 (91·0)  539 (79·9)  195 (28·9)  105 (15·6)  214 (23·1)  250 (27·0)  47 (5·1)  308 (33·3)  91 (9·8)  95/722 (13·2)  134 (14·5)  220 (23·8)  102/890 (11·5) | 0·027  **-**  0·112  -  0·520  0·359  0·818  0·669  0·947  0·533  0·021  0·654  **0·0005**  0·029  **0·006**  0·012  **< 0·0001** |

**Legend:**

^1^ Of parous females
^2^ Lower back pain, fibromyalgia, chronic fatigue syndrome, headache (including migraine), joint hypermobility syndrome (≥2)
^a^ Patients with coexistent symptoms and those with functional constipation in isolation
^b^ ­*p* value represents the statistical difference between patients with faecal incontinence in isolation and reclassified patients(^a^), according to the Rome IV core criteria
IQR = interquartile range
Denominators indicate variables with missing values

**Supplementary Table 2B. Symptoms in patients referred for symptoms of faecal incontinence in isolation, reclassified according to Rome IV core criteria.**

|  | Referred for faecal incontinence in isolation  *N* = 1,640 | Reclassification according to Rome IV core criteria | | | | *p* value^b^ |
| --- | --- | --- | --- | --- | --- | --- |
|  |  | Faecal incontinence  *N* = 714 (43·5%) | Coexistent symptoms  *N* = 765 (46·6%) | Functional constipation  *N* = 161 (9·8%) | Reclassified^a^  *N* = 926 (56·5%) |  |
| **Faecal incontinence** (%) | | | | | | |
| Combined solid and liquid stool  Duration of symptoms (> 5 years)  Type  Urge  Passive  Post-defaecation  Cough  Mixed  Flatus incontinence  Frequency > monthly  Duration of symptoms (> 5 years)  Pads/plugs  Constipation medication (e.g. Loperamide)  Urgency  Poor discrimination between stool and flatus | 469/1,468 (31·9)  204/1,441 (14·2)  131/1,441 (9·1)  114/1,441 (7·9)  20/1,441 (1·4)  972/1,441 (67·5)  1,170 (71·3)  398/1,097 (36·3)  877 (53·5)  367 (22·4)  1,277 (77·9)  855/1,596 (53·6) | 215/711 (30·2)  132/696 (19·0)  65/696 (9·3)  58/696 (8·3)  9/696 (1·3)  432/696 (62·1)  475 (66·5)  141/449 (31·4)  384 (53·8)  173 (24·2)  577 (80·8)  344/697 (49·4) | 254/757 (33·6)  72/745 (9·7)  66/745 (8·9)  56/745 (7·5)  11/745 (1·5)  540/745 (72·5)  602 (78·7)  233/564 (41·3)  466 (60·9)  172 (22·5)  604 (79·0)  452/743 (60·8) | -  -  -  -  -  -  93 (57·8)  24/84 (28·6)  27 (16·8)  22 (13·7)  96 (59·6)  59/156 (37·8) | 254/757 (33·6)  72/745 (9·7)  66/745 (8·9)  56/745 (7·5)  11/745 (1·5)  540/745 (72·5)  695 (75·1)  257/648 (39·7)  493 (53·2)  194 (21·0)  700 (75·6)  511/899 (56·8) | 0·174  **< 0·0001**  0·751  0·574  0·766  **< 0·0001**  **0·0002**  **0·005**  0·827  0·114  0·012  **0·003** |
| **Constipation** (%) | | | | | | |
| Duration of symptoms (>10 years)  Time on lavatory (>10 minutes)  Oral laxative use  Unsuccessful bowel movements (>25%)  Painful defaecation (>25%)  Abdominal pain (>25%)  Bloating (>25%)  **Straining (>25%)**  **Incomplete rectal emptying (>25%)**  **Anorectal blockage (>25%)**  **Manual manoeuvres (>25%)** | 333 (20·3)  335 (20·4)  305/1,539 (19·8)  526 (32·1)  662 (40·4)  872 (53·2)  380/1,602 (23·7)  801 (48·8)  1,227 (74·8)  574 (35·0)  257 (15·7) | -  63 (8·8)  48/681 (7·0)  42 (5·9)  176 (24·7)  312 (43·7)  121/697 (17·4)  55 (7·7)  362 (50·7)  8 (1·1)  3 (0·4) | 231 (30·2)  236 (30·8)  219/707 (31·0)  406 (53·1)  405 (52·9)  477 (62·4)  226/746 (30·3)  617 (80·7)  721 (94·2)  472 (61·7)  215 (28·1) | 47 (29·2)  36 (22·4)  38/151 (25·2)  78 (48·4)  81 (50·3)  83 (51·6)  33/159 (20·8)  129 (80·1)  144 (89·4)  94 (58·4)  39 (24·2) | 278 (30·0)  272 (29·4)  257/858 (30·0)  484 (52·3)  486 (52·5)  560 (60·5)  259/905 (28·6)  746 (80·6)  865 (93·4)  566 (61·1)  254 (27·4) | **< 0·0001**  **< 0·0001**  **< 0·0001**  **< 0·0001**  **< 0·0001**  **< 0·0001**  **< 0·0001**  **< 0·0001**  **< 0·0001**  **< 0·0001**  **< 0·0001** |
| **Prolapse** (%) | | | | | | |
| Feeling of bulge  Blood loss per rectum  Mucous discharge per rectum | 632/1,595 (39·6)  616/1,572 (39·2)  917/1,562 (58·7) | 178/693 (25·7)  205/684 (30·0)  354/684 (51·8) | 387/742 (52·2)  344/734 (46·9)  485/725 (66·9) | 67/160 (41·9)  67/154 (43·5)  78/153 (51·0) | 454/902 (50·3)  411/888 (46·3)  563/878 (64·1) | **< 0·0001**  **< 0·0001**  **< 0·0001** |
| **Other** (%) | | | | | | |
| Bowel frequency  **Infrequent (<3x per week)**  Normal (1 – 2x per 1 – 2 days)  Frequent (≥3x per day)  Stool consistency  **Hard (Bristol 1 – 2)**  Normal (Bristol 3 – 5)  Liquid (Bristol 6 – 7)  Variable  IBS (Rome III criteria) | 200/1,595 (12·5)  614/1,595 (38·5)  781/1,595 (49·0)  121/1,620 (7·5)  405/1,620 (25·0)  261/1,620 (16·1)  833/1,620 (51·4)  221/797 (27·7) | 9/701 (1·3)  288/701 (41·1)  404/701 (57·5)  3/702 (0·4)  229/702 (32·6)  161/702 (22·9)  309/702 (44·0)  66/358 (18·4) | 152/737 (20·6)  257/737 (34·9)  328/737 (44·5)  92/758 (12·1)  143/758 (18·9)  89/758 (11·7)  434/758 (57·3)  132/352 (37·5) | 39/157 (24·8)  69/157 (43·9)  49/157 (31·2)  26/160 (16·3)  33/160 (20·6)  11/160 (6·9)  90/160 (56·3)  23/87 (26·4) | 191/894 (21·4)  326/894 (36·5)  377/894 (42·2)  118/918 (12·9)  176/918 (19·2)  100/918 (10·9)  524/918 (57·1)  155/439 (35·3) | **< 0·0001**  0·060  **< 0·0001**  **< 0·0001**  **< 0·0001**  **< 0·0001**  **< 0·0001**  **< 0·0001** |

**Legend:**

Underlined symptoms are part of the Rome IV diagnostic criteria for functional constipation^1^

^a^ Patients with coexistent symptoms and those with functional constipation in isolation

^b^ ­*p* value represents the statistical difference between patients with faecal incontinence in isolation and reclassified patients(^a^), according to the Rome IV core criteria

Denominators indicate variables with missing values

**Supplementary Table 2C. Proportions of patients with abnormal findings on anorectal physiological testing in patients referred for symptoms of faecal incontinence in isolation, reclassified according to Rome IV core criteria, in those with a minimum of anorectal manometry, rectal sensation testing and endoanal ultrasonography (N = 1,512; 92·2% of the total study sample).**

|  | Referred for faecal incontinence in isolation  *N* = 1,512 | Reclassification according to Rome IV core criteria | | | | *p* value^b^ |
| --- | --- | --- | --- | --- | --- | --- |
|  |  | Faecal incontinence  *N* = 662 (43·8%) | Coexistent symptoms  *N* = 707 (46·8%) | Functional constipation  *N* = 143 (9·5%) | Reclassified^a^  *N* = 850 (56·2%) |  |
| Anorectal manometry^1^ (%)  Normal  Anal hypotension + normal contractility  Anal normotension + hypocontractility  Anal hypotension + hypocontractility | 617 (40·8)  185 (12·2)  371 (24·5)  339 (22·4) | 276 (41·7)  78 (11·8)  157 (23·7)  151 (22·8) | 260 (36·8)  97 (13·7)  181 (25·6)  169 (23·9) | 81 (56·6)  10 (7·0)  33 (23·1)  19 (13·3) | 341 (40·1)  107 (12·6)  214 (25·2)  188 (22·1) | 0·537  0·635  0·655  0·749 |
| Endoanal ultrasonography (%)  *Internal anal sphincter*  Intact  Degenerate/atrophic  Disrupted  Abnormal, focal  *External anal sphincter*  Intact  Degenerate/atrophic  Disrupted  Abnormal, focal | 763 (50·5)  375 (24·8)  345 (22·8)  111 (7·3)  674 (44·6)  141 (9·3)  559 (37·0)  211 (14·0) | 328 (49·6)  165 (24·9)  156 (23·6)  45 (6·8)  294 (44·4)  59 (8·9)  248 (37·5)  89 (13·4) | 347 (49·1)  181 (25·6)  167 (23·6)  56 (7·9)  311 (44·0)  69 (9·8)  268 (37·9)  95 (13·4) | 88 (61·5)  29 (20·3)  22 (15·4)  10 (7·0)  69 (48·3)  13 (9·1)  43 (30·1)  27 (18·9) | 435 (51·2)  210 (24·7)  189 (22·2)  66 (7·8)  380 (44·7)  82 (9·7)  311 (36·6)  122 (14·4) | 0·530  0·922  0·541  0·474  0·909  0·626  0·727  0·613 |
| Rectal sensation to balloon distension^1^ (%)  Normal  Rectal hypersensitivity  Rectal hyposensitivity | 1,253 (82·9)  105 (6·9)  154 (10·2) | 547 (82·6)  53 (8·0)  62 (9·4) | 595 (84·2)  37 (5·2)  75 (10·6) | 111 (77·6)  15 (10·5)  17 (11·9) | 706 (83·1)  52 (6·1)  92 (10·8) | 0·826  0·152  0·930 |
| Whole-gut transit studies (%)  Delayed | 154 (10·2)  31 (1·9) | 50 (7·6)  9 (18·0) | 85 (12·0)  17 (20·0) | 19 (13·3)  5 (26·3) | 104 (12·2)  22 (21·2) | **0·003**  0·648 |
| Defaecography (%)  Functional abnormality  Significant structural abnormality  Intussusception  Obstructing recto-rectal  Recto-anal  Rectocoele  Depth ≥4cm  Depth 2–4cm, symptomatic  Enterocoele  Megarectum  Rectal prolapse  Functional + structural abnormality | 1,294 (85·6)  300 (23·2)  584 (45·1)  149 (11·5)  219 (16·9)  135 (10·4)  68 (5·3)  47 (3·6)  59/455 (13·0)  38 (2·9)  79 (6·1) | 532 (80·4)  124 (23·3)  207 (38·9)  67 (12·6)  91 (17·1)  46 (8·7)  4 (0·8)  9 (1·7)  22/167 (13·2)  7 (1·3)  26 (4·9) | 636 (90·0)  151 (23·7)  312 (49·1)  67 (10·5)  99 (15·6)  71 (11·2)  52 (8·2)  29 (4·6)  30/230 (13·0)  30 (4·7)  47 (7·4) | 126 (88·1)  25 (19·8)  65 (51·6)  15 (11·9)  29 (23·0)  18 (14·3)  12 (9·5)  9 (7·1)  7/58 (8·6)  1 (0·8)  6 (4·8) | 762 (89·7)  176 (23·1)  377 (49·5)  82 (10·8)  128 (16·8)  89 (11·7)  64 (8·4)  38 (5·0)  37/288 (12·8)  31 (4·1)  53 (7·0) | **< 0·0001**  0·929  **0·0002**  0·310  0·885  0·079  **< 0·0001**  **0·002**  0·920  **0·004**  0·126 |

**Legend:**

^1^ Diagnostic classification based on the London classification for disorders of anorectal function^9^

^a^ Patients with coexistent symptoms and those with functional constipation in isolation

^b^ ­*p* value represents the statistical difference between patients with faecal incontinence in isolation and reclassified patients(^a^), according to the Rome IV core criteria

**Bowel questionnaire**

From the bowel questionnaire, the following validated scoring systems related to constipation and faecal incontinence could be derived: Rome IV core criteria for functional constipation applied *post hoc* (during >25% of defaecations: 1) straining; 2) lumpy or hard stool; 3) feeling of incomplete evacuation; 4) feeling of anorectal obstruction; 5) manual manoeuvres; 6) < 3 defaecations per week),^1^ Cleveland Clinic constipation score (range 0–30),^2^ Rome IV criteria for faecal incontinence, also applied *post hoc* (> monthly episodes),^3^ and St Marks incontinence score (range 0–24).^4^ Besides these scoring systems, the questionnaire also incorporated other (bowel related) scoring systems and questions, including the Rome III criteria for irritable bowel syndrome,^5^ and the joint hypermobility syndrome validated 5-point screening questionnaire.^6^ Relevant surgical procedures were classified as anal/perineal, abdominal/bowel, rectal or pelvic (including hysterectomy). Vaginal deliveries associated with an episiotomy/perineal tear were defined as traumatic; forceps/Ventouse-assisted deliveries were classified as instrumental.

**Anorectal physiology measurements**

*Anorectal manometry*

Studies prior to 2013 were performed with a station pull-through technique, using a water-perfused manometric system (Medical Measurement Systems [MMS], Enschede, The Netherlands). Normal values were based on a dataset of 82 healthy asymptomatic individuals assessed previously within the GI Physiology Unit.^7^ Studies performed between 2013–2016 were performed with a high-resolution anorectal manometry system (Solar GIHRM v9.1; MMS), utilising a solid-state catheter incorporating 12 micro transducers (UniTip: UniSensor AG, Attikon, Switzerland). Normal values were based on our previously published work.^8^ Anal hypotonia was defined as a resting tone below normal limits; anal hypocontractility was diagnosed if maximum voluntary incremental squeeze pressure was below normal limits.^9^

*Rectal sensation to balloon distension*

A detailed prescription of the departmental protocol has been published previously.^10^ Rectal hypersensitivity was defined by the maximum tolerable volume below normal limits. Hyposensitivity was diagnosed when ≥2 rectal sensory thresholds were above normal limits.^11^ Levels of normality were based on our previously published work.^10^

*Endoanal ultrasonography*

Two dimensional cross-sectional axial images of the anal canal from the level of the puborectalis muscle until the anal verge were acquired using a 13 MHz transducer (BK Medical 2101, Berkshire, United Kingdom). Both the internal and external anal sphincter were classified as intact or abnormal (disrupted, degenerate/atrophic, or focally abnormal). Sphincter disruption was defined by a discontinuity of the muscle ring or by loss of muscle architecture. Degeneration/atrophy was diagnosed if the anal sphincter was thin or poorly defined, often with increased echogenicity. Focal abnormalities were defined by scarring, thinning or an area of mixed echogenicity.

*Radio-opaque whole-gut transit studies*

Delayed whole-gut transit time was diagnosed if >20% of 50 ingested markers were retained at 100 hours after ingestion, as visualised on a plain abdominal radiograph.^12, 13^

*Defaecography*

The departmental protocol has been published in detail previously.^14^ A rectal evacuation disorder was defined as being secondary to functional and/or structural abnormalities.^15^ A functional abnormality was diagnosed by incomplete (<60% instilled contrast) or protracted (>150 seconds) evacuation allied to poor opening of the anorectal angle, poor relaxation of the anal canal or poor expulsive force generated.^14^ Structural abnormalities comprised significant intussusception (obstructing recto-rectal [Oxford grade I – II] or recto-anal [Oxford grade III–IV]),^16^ rectocoele (depth >4cm or 2-4 cm allied to symptoms of obstructed defaecation),^14, 15^ enterocoele,^15^ megarectum (mid rectal diameter >8·1 cm in men and >6·9 cm in women; routinely measured from 2013 onwards)^14^ and external rectal prolapse (Oxford grade V).^16^

**REFERENCES SUPPLEMENTARY DOCUMENT**

1. Mearin F, Lacy BE, Chang L, et al. Bowel Disorders. *Gastroenterology* 2016;**150**:1393-1407.

2. Agachan F, Chen T, Pfeifer J, et al. A constipation scoring system to simplify evaluation and management of constipated patients. *Dis Colon Rectum* 1996;**39**:681-5.

3. Rao SS, Bharucha AE, Chiarioni G, et al. Functional Anorectal Disorders. *Gastroenterology* 2016;**150**:1430-1442.

4. Vaizey CJ, Carapeti E, Cahill JA, et al. Prospective comparison of faecal incontinence grading systems. *Gut* 1999;**44**:77-80.

5. Longstreth GF, Thompson WG, Chey WD, et al. Functional bowel disorders. *Gastroenterology* 2006;**130**:1480-91.

6. Hakim AJ, Grahame R. A simple questionnaire to detect hypermobility: an adjunct to the assessment of patients with diffuse musculoskeletal pain. *Int J Clin Pract* 2003;**57**:163-6.

7. Vasudevan SP. Rectal hyposensitivity: clinical and physiological impact on patients with chronic constipation. <https://qmro.qmul.ac.uk/xmlui/handle/123456789/8842> 2014.

8. Carrington EV, Brokjaer A, Craven H, et al. Traditional measures of normal anal sphincter function using high-resolution anorectal manometry (HRAM) in 115 healthy volunteers. *Neurogastroenterol Motil* 2014;**26**:625-35.

9. Carrington EV, Heinrich H, Knowles CH, et al. The international anorectal physiology working group (IAPWG) recommendations: Standardized testing protocol and the London classification for disorders of anorectal function. *Neurogastroenterol Motil* 2019:e13679.

10. Townsend DC, Carrington EV, Grossi U, et al. Pathophysiology of fecal incontinence differs between men and women: a case-matched study in 200 patients. *Neurogastroenterol Motil* 2016;**28**:1580-8.

11. Carrington EV, Heinrich H, Knowles CH, et al. The international anorectal physiology working group (IAPWG) recommendations: Standardized testing protocol and the London classification for disorders of anorectal function. *Neurogastroenterol Motil* 2020;32:e13679.

12. Hinton JM, Lennard-Jones JE, Young AC. A ne method for studying gut transit times using radioopaque markers. *Gut* 1969;**10**:842-7.

13. Roberts JP, Newell MS, Deeks JJ, et al. Oral [111In]DTPA scintigraphic assessment of colonic transit in constipated subjects. *Dig Dis Sci* 1993;**38**:1032-9.

14. Palit S, Bhan C, Lunniss PJ, et al. Evacuation proctography: a reappraisal of normal variability. *Colorectal Dis* 2014;**16**:538-46.

15. Grossi U, Di Tanna GL, Heinrich H, et al. Systematic review with meta-analysis: defecography should be a first-line diagnostic modality in patients with refractory constipation. *Aliment Pharmacol Ther* 2018;**48**:1186-1201.

16. Adusumilli S, Gosselink M, Fourie S, et al. Does the presence of a high grade internal rectal prolapse affect the outcome of pelvic floor retraining in patients with faecal incontinence or obstructed defaecation? *Colorectal Disease* 2013;**15**:e680-e685.
